# D-carvone induced ROS mediated apoptotic cell death in human leukemic cell lines (Molt-4)

**DOI:** 10.6026/97320630017171

**Published:** 2021-01-31

**Authors:** Petchi Iyappan, M Devi Bala, M Sureshkumar, Vishnu Priya Veeraraghavan, Arulselvan Palanisamy

**Affiliations:** 1 Senior Lecturer, Faculty of Medicine, Bioscience and Nursing, School of Bioscience, Mahsa University, Saujana Putra Campus, Jalan SP2, Bandar Saujana Putra, 42610, Jenjarom, Selangor, Malaysia; 2 Research Scholar, Muthayammal College of Arts & Science (A Unit of VANETRA Group), Rasipuram, 637408, Namakkal, Tamilnadu, India; 3 Department of Zoology & Biotechnology, Muthayammal College of Arts & Science (A Unit of VANETRA Group), Rasipuram, 637408, Namakkal, Tamilnadu, India; 4 Department of Biochemistry, Saveetha Dental College, Saveetha Institute of Medical and Technical Sciences, Saveetha University, Chennai - 600 077; 5 Adjunct Associate Professor,Muthayammal Centre for Advanced Research (MCAR), Muthayammal College of Arts & Science (A Unit of VANETRA Group),Rasipuram, 637408, Namakkal, Tamilnadu, India

**Keywords:** D-Carvone, Human leukemic cells, MOLT-4 cells, antioxidants, apoptosis, mitochondrial membrane potential

## Abstract

The immature lymphoid cells with chromosomal structural and numerical abnormalities cause the acute lymphoblastic leukemia (ALL). This hematologic disorder constitutes about 25% of cancer prognosis among children and adolescents. D-Carvone, a monocyclic monoterpene obtained from the essential oils extracted from plants is reported to possess the various biological activities. The present study was aimed to investigate the anticancer potential of D-Carvone against the human leukemic Molt-4 cells. The cytotoxicity of DCarvone was analyzed by MTT assay. The level of lipid peroxidation and antioxidants were determined. The intracellular ROS, MMP and apoptosis were demonstrated by fluorescent staining techniques. The MTT assay revealed that the D-Carvone treatment suppressed the viability of Molt-4 cells and the IC_50_ was determined at 20 µM/ml. The D-Carvone treatment was increased the oxidative stress and reduced the level of antioxidants in the Molt-4 cell lines. The increased intracellular ROS, apoptotic cell death, and diminished MMP was noted in the D-Carvone treatment. In the Molt-4 cells, D-carvone induced the apoptosis in a time and dose dependent manner by the activation of caspases-8, -9 and -3. Thus, data provide insights for the clinical application of D-Carvone in the treatment of blood cancer Molt-4 cells. Our study suggests the therapeutic potential D-Carvone for the treatment of leukemia in future.

## Background:

The resources for treatment of cancer are minimal in developing nations [1]. Acute lymphoblastic leukemia (ALL) is a heterogeneous hematological malignancy caused by lymphoid cell proliferation and abnormal differentiation in bone marrow, peripheral blood and various other organs [2]. The high degree of heterogeneity is attributed to diverse genetic alterations that were acquired over a period of time. These alterations include both numerical and structural rearrangement of chromosomes, gain and loss of genomic regions and single nucleotide alterations. Approximately 75 to 80 % children develop ALL compared to adults. Anti-leukemic drugs and improved multimodality treatment has increased the 5 year survival rate of children with ALL above 85 % in developed countries [3] but majority take a turn for the worse within 2 years of diagnosis resulting in a five year survival rate is reduced to 10 % [4]. In the view of the above facts investigation of therapeutic substances that are effective in reducing the carcinogenicity of mutagens gained importance [5-7]. Antioxidants are important for their protective role followed by the free radicals induced damages, which may lead to several cancers including blood cancer [8]. Most of the chemotherapeutic agents currently employed are directly or indirectly derived from the natural sources. Among these natural sources secondary metabolites from plants and their derivatives have been proved their potentials as effective anticancer drugs [9,10]. Monoterpenes present in plant essential oils contribute to the aroma of plants [11,12]. D-carvone is one such monoterpenic ketones that is present in various essential oils extracted from plants and has been utilized in food and pharmaceutical industry [13,14]. Literature also reports that this bioactive compound possesses antimicrobial, antioxidant, antitumor and anticonvulsant properties [15-17]. D-carvone also possessed the neuroprotective [18], anticancer [19,20], antiarthritis [21], and anti-ulcerative colitis [22] activities. Therefore, it is of interest to document data on the D-carvone ROS mediated apoptotic cell death in human leukemic cell lines (Molt-4).

## Materials and methods:

### Chemicals and reagents:

**Figure 1:**
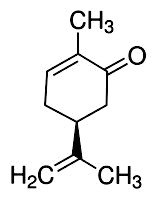
Chemical structure of D-carvone.

D-carvone (> 96% purity) (Figure 1) was obtained at Sigma Aldrich (USA). Fetal bovine serum (FBS), Dulbecco's modified eagle medium (DMEM), penicillin (100 units/ml), streptomycin (100 g/ml), trypsin-EDTA, phosphate buffered saline (PBS), 2', 7'-dichlorofluorescin diacetate (DCFH-DA), acridine orange (AO)/ethidium bromide (EB), 3-(4,5-Dimethylthiazol-2-yl)-2,5-diphenyltetrazolium bromide (MTT), rhodamine-123 (Rh-123) and propidium iodide (PI) staining were obtained from HiMedia (USA).

### Cell culture, proliferation and treatments:

Human ALL (Molt-4) cells were purchased from the ATCC, USA. Cells were developed in DMEM medium supplemented with heat inactivated FBS (10%) and antibiotics and maintained in the CO_2_ incubator at 37°C in an atmosphere of air (95%) and CO_2_ (5%) with 98% humidity.

### MTT cytotoxicity assay:

The cytotoxicity effects of D-carvone against the Molt-4 cells were analyzed by the method of Mosmann et al. (1983) [23]. Briefly, 6 x 103 cells per well were seeded in 96 well plate and were treated with different concentrations of D-carvone (5 µM to 30 µM) for 24 h. 20 µl of MTT dye (2.5 mg/ml) was added and incubated for 3 h before the termination of the experiment. After that, the culture plate was incubated for 4 h at 37°C and formulated formazan crystals were dissolved by the addition of 150 µl of DMSO. Finally, the absorbance was measured at 570 nm with reference wavelength at 620 nm. The percentage of cell viability was calculated at 50% inhibitory concentration (IC50) was determined.

### Measurement of intracellular ROS:

The intracellular accumulation of ROS was monitored using DCFH-DA staining technique. Deacetylation within the cell leads to the binding of dye with intracellular radicals, generated in a quantitative manner and it is converted into its fluorescent product DCF. Molt-4 cells treated with D-carvone for 24 h were harvested and re-suspended in PBS (pH 7.4). DCFH-DA solution (10 µM) was added to 2 x 105 ml of cell suspension. The mixture was incubated at 37°C for 30 min. Cells were then washed twice and re-suspended in PBS. The fluorescence intensity was measured spectroflurometrically with excitation and wavelengths ranging from 485 nm and 530 nm respectively.

### Measurement of MMP:

Molt-4 cells were seeded in 6-wellplate and incubated with different concentrations of D-carvone (15 and 20 µM/ml) for 24 h. Rh-123 is a fluorescent probe used to estimate the depolarization of MMP. Rh-123 dye was added and incubated for 30 min at 37°C. After incubation the cells were washed with PBS and observed under fluorescence microscope (Labomed, USA). Fluorescence intensity of the captured images was analyzed by Image J software. Fluorescence intensity of the captured images was taken using a blue filter (450–490 nm).

### Observation of morphological and nuclear changes AO/EB staining

Briefly, the AO/EB (AO: 100µg/ml, EB: 100µg/ml) stain solution was added to D-carvone (15 and 20 µM/ml) treated Molt-4 cells placed in a cover slip. Placing the cover slip over it spread the dye. The stained slides were then incubated at room temperature for 5 min. The apoptotic cells were visualized for green fluorescence, which was counted using an upright fluorescent microscope at 40x magnification.

### PI staining:

After treatment with the (15 and 20 µM/ml) of D-Carvone for 24h, the traces of medium and serum were removed from Molt-4 cells and cleansed with PBS. The cells were permeabilized using 50µl acetone and methanol in 1:1 ratio at -20°C for 10 min. Then 10µl of PI was added and spread by placing the cover slip over it and incubated at 37°C for 30 min in dark. Finally the PI stained cells were observed under the fluorescence microscope.

### Estimation of caspases -8, -9 and -3 activity:

Molt-4 cells were incubated with D-carvone for 24 h. The activities of the caspases were carried out using colorimetric protease assay (Invitrogen, USA) following the manufacturer's protocol. Each kit contains a specific substrate: IETD (Ile-GluThr-Asp), LEHD (Leu-Glu-His-Asp) and Ac-DEVD (acetyl-AspGlu-Val-Asp) for caspases -8, -9 and -3, respectively. Such substrates are labeled to the chromophore p-nitroanilide (pNA), which is released when they are cleaved by activated caspases and measured at 405 nm in a spectrophotometer (Biotek Instruments EL800, USA).

### Biochemical analysis:

The Molt-4 cells were treated with D-carvone and harvested for the following biochemical investigations. The thiobarbituric acid reactive substances (TBARS) were evaluated through the Ohkawa et al. (1979) [24] technique and enzymatic antioxidant activities such as catalase (CAT) was examines by the way of Sinha (1972) [25], superoxide dismutase (SOD) were demonstrated via a method of Kakkar et al. (1984) [26] and glutathione (GSH) were investigated illustrated through way of Moron et al. (1979) [27] respectively.

### Statistical Analysis:

Data were illustrated as mean ± SD of triplicate measurement. Statistical evaluations were assessed using the SPSS software. Significance level was calculated by using one-way ANOVA followed by DMRT test. Results are considered as statistically significant if p < 0.05.

## Results:

### Effect of D-carvone on cell viability of Molt-4 cell lines:

**Figure 2:**
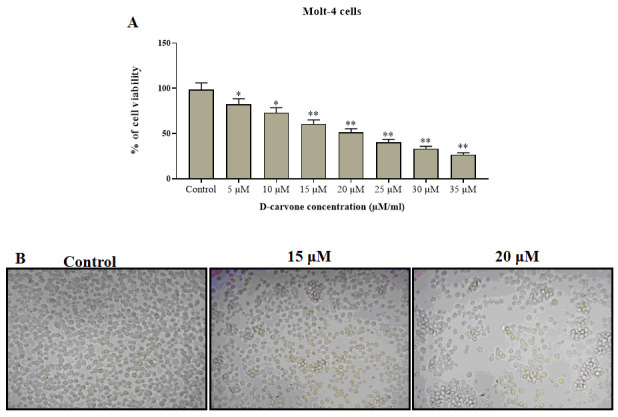
The cytotoxic effect of D-carvone on MOLT-4 cells. A) Cancer cells were cultured with D-carvone at various concentrations for 24 h and cell viability was evaluated by MTT assay. B) D-carvone caused a morphology change of Molt-4 cells. Phase contrast images of Molt-4 cells treated with increasing concentrations of D-carvone for 24 h. The results are mean ± SD of triplicates from three independent experiments, *p < 0.05 versus control.

Figure 2 presents the cytotoxic effects of D-Carvone on the cell viability of Molt-4 cells. The D-Carvone treated Molt-4 cells were demonstrated the notable cytotoxicity by the dose dependent manner. The morphological alterations in Molt-4 cells were observed through bright field phase contrast microscope (Figure 2A). Significant loss of cell viability and morphological alterations were witnessed with a 15 µM of D-carvone treatment and an increase in concentration resulted in further morphological alterations. D-carvone notably decreased the viability of Molt-4 cells at 24 h with IC50 value of 20 µM (Figure 2B). Hence for the further studies 15 µM and 20 µM/ml of D-carvone concentrations were considered.

### Measurement intracellular ROS level in D-carvone:

**Figure 3:**
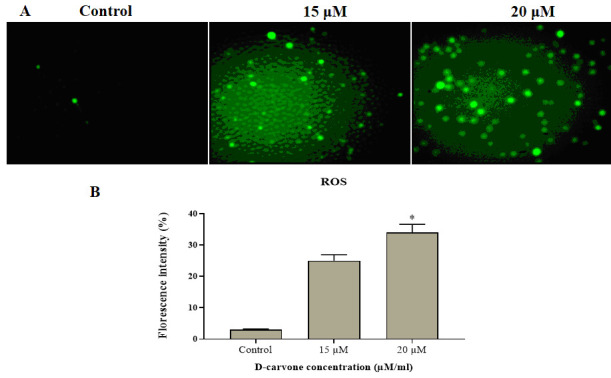
DCFH-DA staining test for examination of D-carvone induced intracellular ROS level. (A) Healthy Molt-4 cancerous cells without fluorescence (no ROS), D-carvone (15 µM/ml) treated cells with less accumulated ROS, and D-carvone (20 µM/ml) treated cells with increased accumulated of ROS. B) Shows the mean fluorescent intensity of control and D-carvone treated Molt-4 cells. The results are mean ± SD of triplicates from three independent experiments, *p < 0.05 versus control.

ROS generation in Molt-4 cells exposed to different concentrations of D-carvone (15 µM and 20 µM/ml) was accessed by using DCFH-DA staining (Figure 3). Level of ROS was observed by the intense green fluorescence (Figure 3). D-carvone (15 µM/ml) treated Molt-4 cells were depicted weak background fluorescence, while treatment with high dose D-carvone (20 µM/ml) showed bright green fluorescence, which indicates the increased ROS level. The ROS observed in (Figure 3) Molt-4 cells exposed with 15 µM and 20 µM/ml concentrations were moderately changed (p < 0.05) 24 and 35% respectively, when related with untreated cells in a dose dependent manner.

### Measurement of MMP level in D-carvone:

**Figure 4:**
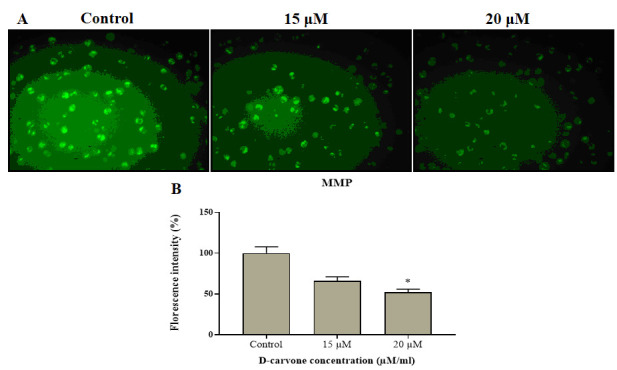
Mitochondrial staining using Rh-123 of human blood cancer cells treated with D-carvone. Molt-4 cells were treated with different concentration D-carvone for 24 h, stained with Rh-123 and the MMP patterns of Molt-4 cells were examined. Results the decrease of green fluorescence indicates MMP in a concentration manner were analyzed by fluorescent microscope. In the fluorescent image shows control (Rh-123 accumulation); D-carvone 15 and 20 µM/ml (No Rh-123 accumulation). B) Shows the mean fluorescent intensity of control and D-carvone treated Molt-4 cells. The results are mean ± SD of triplicates from three independent experiments, *p < 0.05 versus control.

To monitor the MMP level in the D-Carvone (15 µM and 20 µM/ml) treated Molt-4 cells, the Rh-123 staining was executed and the result was illustrated in the [Figure 4]. [Figure 4 illustrated that the untreated control cells displayed an intense green fluorescence, alternatively, the Molt-4 cells treated with the D-Carvone was demonstrated the dull green fluorescence, which evidencing the declined MMP in the Molt-4 cells. This result was proved that the D-Carvone has the capacity to reduce the MMP of Molt-4 cells (Figure 4B).

### Effect of D-carvone induced apoptotic cell death in Molt-4 cells:

**Figure 5:**
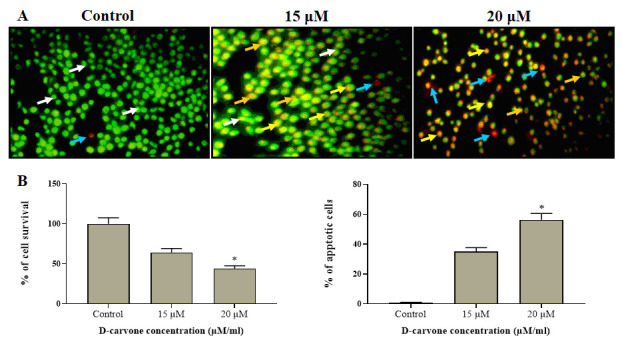
Effect of D-carvone induces apoptotic cell death in Molt-4 cells. (A) Blood cancer cells treated within control and D-carvone at various concentration manners at 24 h, stained with AO/EB and then examined through fluorescence microscope. Green florescence (White arrow); apoptotic bodies (Orange arrow); apoptotic cells (Blue arrow); necrotic cells (Yellow arrow). D-carvone activated apoptosis via producing ROS and disturbance of MMP. (B) Percentage of apoptotic cells was quantified by scoring apoptotic and viable cells. The values are given as mean ± SD of three experiments in each group ANOVA followed by DMRT. Asterisks indicate statistically different from control * p < 0.05.

The apoptotic cell death in the D-Carvone (15 µM and 20 µM) treated Molt-4 cells was investigated by dual (AO/EtBr) staining technique. Figure 5A revealed that the untreated control cells displayed AO stained green fluorescence; interestingly, the D-Carvone treated Molt-4 cells were revealed the intense EtBr stained orange fluorescence that demonstrating the apoptotic cells. Hence, it was clear that the D-Carvone has the potential to stimulate apoptosis in blood cancer cells. Both concentrations (15 and 20µg) of the D-Carvone has enthused the apoptosis in the human blood cancer Molt-4 (Figure 5).

### Effect of D-carvone induced apoptosis in Molt-4 cells:

**Figure 6:**
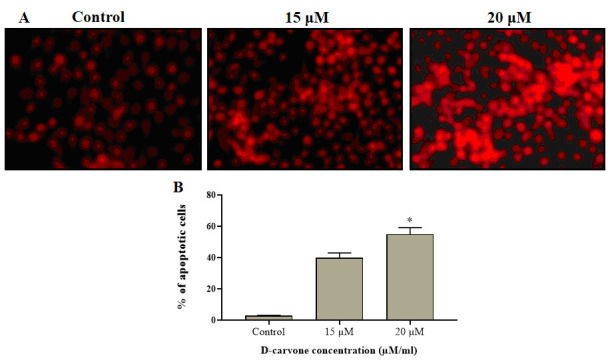
Effect of D-carvone induces apoptotic cell death in Molt-4 cells.

The PI was executed to differentiate the necrotic cells from viable cells. [Figure 6] illustrating the intense red fluorescence in the D-Carvone treated Molt-4 cells than the untreated control cells, which proves the occurrence of necrotic cells. The strong red fluorescence was indicating the increased number of apoptotic or necrotic cells. Hence it was clear that the D-Carvone treatment could stimulate the apoptotic cell necrosis in the Molt-4 cells.

### Effect of D-carvone on MDA and antioxidant enzymes of Molt-4 cells:

**Figure 7:**
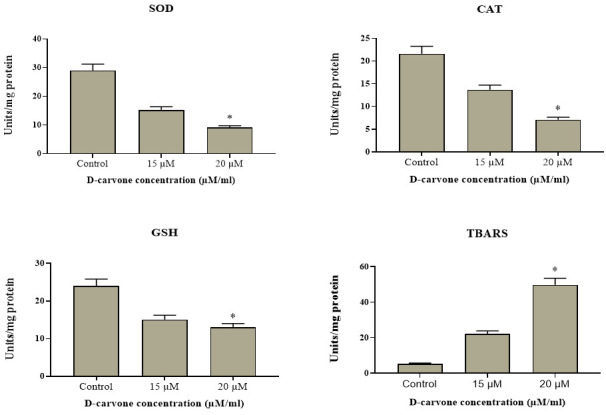
D-carvone activated LPO and modulates cellular antioxidant activities in Molt-4 cells. The results are mean ± SD of triplicates from three independent experiments, *p < 0.05 versus control.

Figure 7 shows the lipid peroxidation and antioxidant levels in control and D-carvone treated blood cancer Molt-4 cell line. We found an augmented level of TBARS and decreased status of SOD, GSH and CAT enzymes in the D-carvone (15 and 20 µM/ml) treated Molt-4 cells when compared to control cells. The status of LPO and antioxidants level is a well-known biomarker for the oxidative stress in blood cancer cells. This result proved that the D-Carvone increased the oxidative stress in the Molt-4 cells, thereby leads to oxidative cell damages.

### Estimation of caspase -3, -9 and -8 activities in Molt-4 cells:

**Figure 8:**
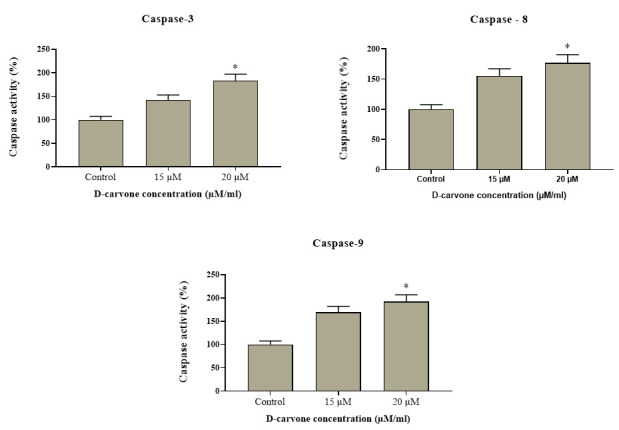
Effect of D-Carvone on caspase -3, -9 and -8 activities in Molt-4 cell. The ALL cells (Molt-4) were treated with D-carvone for 24 h and then harvested. The protein levels were observed through ELISA technique. The results are mean ± SD of triplicates from three independent experiments, *p < 0.05 versus control.

Figure 8 shows that the pro-apoptotic protein expression of control and D-carvone treated blood cancer Molt-4 cells. The control cells showed the down-regulated expression of caspase-3, -8 and -9. Our results shows that the D-Carvone treated Molt-4 cells were revealed the up-regulated expressions of caspase-3, -9, and -8 when compared to the control. D-carvone significantly (p < 0.05) augmented the levels of pro-apoptotic markers expression. This result indicates that D-carvone induced the expression of pro-apoptotic markers in Molt-4 cells.

## Discussion:

Nutrients have a very important role in maintaining normal health. Dietary antioxidants are potential adjuvant in cancer therapy since they are capable of inducing apoptosis in the cancer cells [28]. Apoptosis or programmed cell death is an essential mechanism for the development and homeostasis of multicellular organism for eliminating unwanted cells [29]. Failure or inefficient apoptosis is an important factor of tumorigenicity and induction of apoptosis is the target for cancer therapy [30]. The major phytochemicals such as flavonoids, terpenoids, carotenoids and selenium were reported for their anticancer property against numerous cancers [32]. Previous literatures demonstrated the anti-carcinogenic properties of several monoterpenes in experimental models such as liver, melanoma, breast and prostate cancer [31]. Carvone a monoterpene ketone is found predominantly in essential oils of spearmint and caraway is used as an odorant and flavor accompaniment and forms a common ingredient in human diet [33,34]. Literatures report the anti-tumor effect of carvone against tumor cell lines like MCF-7 and HT-29 [36] and HL-60 [37 - check with authors - see PDF version]. Anti-carcinogenic properties of antioxidants were reported in several epidemiological studies and the dietary intake of antioxidants reduces the risks of cancer [38]. The antioxidant capacity of cyano-carvone was reported in mice hippocampus [39]. The results depicted that concentration of 25, 50, and 75 mg/kg of cyano-carvone effectively decreased the level of LPO. DPPH and ORAC assay were employed for the determination of antioxidant activity of carvone [36]. The strong antioxidant activity of S-carvone was reported by Elmastas et al. 2006 [40]. Similarly, the antioxidant capacity of carvone and flavonoids were reported by Saghir et al. 2012 [41]. Carvone above the concentration of 100 mg/mL was found to significantly reduce the cell viability in N2a neuroblastoma cell lines [15]. Literature from earlier studies revealed that monoterpenes such as D-limone, α- pinene, linalool and tylosin had cytotoxic effects [42,43]. Carvone monotypes were reported for a dose-dependent cytotoxic activity against human cervix epithelioid carcinoma cells (HeLa cell line ATCC and CCL-2) [44]. Similarly Hep-2 cells viability and proliferation was inhibited by S (+)-carvone [45]. In contrary, anti-proliferative effect on metastatic B16F-10 melanoma cells by carvone was reported [46]. Though the exact mechanism of cellular toxicity was not known, the oxidative stress might have played a crucial role in the cellular toxicity of carvone. From the results obtained from cell viability assay, it was clearly evident that D-carvone induced cell death in Molt-4 cell lines. In order to understand the mechanism of cell death by D-carvone, staining such as AO/EB and PI were performed to observe the morphological changes in relation to apoptosis. The result showed the chromatin condensation within the nucleus and the formation of apoptotic bodies. Similarly, the AO/EB staining revealed the apoptotic nuclear changes in human colon carcinoma cells (HCT-116) induced by methanolic extract of leaves and fruits of Ligustrum vulgare L [46]. Terpinen-4-ol induced nuclear condensation of Molt-4 leukemic cells was evident with annexin-V-FLUOS staining [47]. Our findings from this study coincide with the above findings. MMP was investigated to understand the mechanism of apoptosis in D-carvone treated Molt-4 cell lines. The control cells were found with intense green fluorescence, which indicated that there were no changes in mitochondrial transmembrane potential. But the cells treated with D-carvone showed loss of green fluorescence due to depolarization of mitochondrial membrane. The effect of D-carvone on the MMP of HT-29 and SW480 colon cancer cell lines with similar observation to the present study was reported [48]. In the non-apoptotic cells the dye were accumulated within the mitochondria and thus exhibiting a bright green color. Decreased accumulation of the dye in the mitochondrial indicates the collapse in the MMP. This may be correlated with high ROS generation. ROS triggers the apoptotic signaling by inducing depolarization of mitochondrial membrane, which results in, increased LPO by-products (TBARS) and decreased activity of antioxidant enzymes (SOD and CAT). These effects are attributes to D-carvone induced ROS generation. The result from the present study was in concordance with earlier studies on colon cancer cells and Hela cells [49]. In order to elucidate the levels of antioxidant enzyme status on the Molt-4 cell lines, the activities of SOD, CAT, GSH and MDA contents were measured. The possible antitumor and antioxidant activity of D-carvone in Molt-4 cell lines were evaluated by measuring the endogenous antioxidant levels. The high accumulation of reactive oxygen species during the process of carcinogenesis may play an important role in causing oxidative damage. Hence there might be an increase or decrease in the antioxidant enzymes. The present study revealed that the activities of SOD and CAT was higher in the untreated MOLT-4 cells (control) compared to D-carvone treated Molt-4 cell lines. A similar observation of higher activity of SOD and CAT were reported in hepatoma (HepG-2) cell lines [44]. The increase in activity of SOD and CAT was reported to be 2 fold and 4.3 fold respectively [50, 51]. These findings were in agreement with our present results that SOD and CAT had increased activity in Molt-4 cells compared to D-carvone treated cell lines. GSH content was found decreased and TBARS content was found increased in D-carvone treated MOLT-4 cell lines. Low levels of GSH were observed in chronic alcoholic liver disease and liver cancer. Such observation of low GSH could be due to alterations in defense system in tumor cells [50]. The study revealed that the anti-tumor activity of D-carvone might influence the status of antioxidant enzymes in Molt-4 cells. The Higher activities of SOD and CAT was reduced significantly with the treatment of D-carvone, Thus D-carvone might have played the role of antioxidants such as SOD and CAT in eliminating the superoxide radicals and accumulation of H2O2 in Molt-4 cell lines. The activation of caspase protein family begins with caspase-3 that initiates apoptosis by activating caspase-8 and -9 [52, 53]. Down regulation of caspase 3 and 9 was reported in DMBA induced skin cancer. D-carvone 20 mg/kg of bw, was recommended as optimal dose for DMBA treated skin cancer in mice [17]. Furthermore, the effect of D-carvone pre-treatment on the expression of the apoptosis-related proteins was determined in our data. As shown in Figure 7, D-carvone pre-treatment abrogated cytochrome c release, as well as the activation of caspase-3, -8 and -9. In agreement with the preceding results of AO/EB and PI staining, these findings indicated that the cytotoxic effect of D-carvone in Molt-4 cells is mediated through apoptotic induction, as well as mitochondrial dysfunction involved in ROS production.

## Conclusion

We document data on the D-carvone induced ROS mediated apoptotic cell death in human leukemic cell lines (Molt-4) for further consideration.
